# Genetic and clinical features of pediatric-onset hereditary spastic paraplegia: a single-center study in Japan

**DOI:** 10.3389/fneur.2023.1085228

**Published:** 2023-05-12

**Authors:** Azusa Ikeda, Tatsuro Kumaki, Yu Tsuyusaki, Megumi Tsuji, Yumi Enomoto, Atsushi Fujita, Hirotomo Saitsu, Naomichi Matsumoto, Kenji Kurosawa, Tomohide Goto

**Affiliations:** ^1^Department of Neurology, Kanagawa Children’s Medical Center, Yokohama, Japan; ^2^Division of Medical Genetics, Kanagawa Children’s Medical Center, Yokohama, Japan; ^3^Clinical Research Institute, Kanagawa Children's Medical Center, Yokohama, Japan; ^4^Department of Human Genetics, Yokohama City University Graduate School of Medicine, Yokohama, Japan; ^5^Department of Biochemistry, Hamamatsu University School of Medicine, Hamamatsu, Japan

**Keywords:** hereditary spastic paraplegia, pediatric-onset hereditary spastic paraplegia, genetic sequencing, diagnostic yield, sporadic

## Abstract

**Background and purpose:**

Hereditary spastic paraplegias (HSPs) are a set of heterogeneous neurodegenerative disorders characterized by bilateral lower limb spasticity. They may present from infancy onwards at any time. Although next-generation sequencing has allowed the identification of many causative genes, little is known about which genes are specifically associated with pediatric-onset variants.

**Methods:**

This study retrospectively evaluated the genetic analyses, family history clinical courses, magnetic resonance imaging (MRI) findings, and electrophysiologic findings of patients diagnosed with HSP in childhood at a tertiary pediatric hospital in Japan. Genetic analyses were performed using direct sequencing, disease-associated panels, and whole-exome sequencing.

**Results:**

Of the 37 patients included, 14 had a family history of HSP and 23 had a sporadic form of the disease. In 20 patients, HSP was the pure type, whereas the remaining 17 patients had complex types of HSP. Genetic data were available for 11 of the pure-type patients and 16 of those with complex types. Of these, genetic diagnoses were possible in 5 (45%) of the pure-type and 13 (81%) of the complex-type patients. *SPAST* variants were found in five children, *KIF1A* variants in four, *ALS2* variants in three, *SACS* and *L1CAM* variants in two each, and an *ATL1* variant in one. One child had a 10p15.3p13 duplication. Four patients with pure-type HSPs had *SPAST* variants and one had an *ALT1* variant. The *KIF1A*, *ALS2*, *SACS*, and *L1CAM* variants and the 10p15.3p13 duplication were seen in children with complex-type HSPs, with just one complex-type patient having a *SPAST* variant. The identification of brain abnormalities on MRI was significantly more common among children with complex-type (11 [69%] of 16) than pure-type HSPs (one [5%] of 19) (*p* < 0.001). Scores on the modified Rankin Scale for Neurologic Disability were also significantly higher among children with complex-type compared with pure-type HSPs (3.5 ± 1.0 vs. 2.1 ± 0.9, *p* < 0.001).

**Conclusion:**

Pediatric-onset HSP was found to be sporadic and genetic in a substantial proportion of patients. The causative gene patterns differed between children with pure-type and complex-type HSPs. The causative roles of *SPAST* and *KIF1A* variants in pure-type and complex-type HSPs, respectively, should be explored further.

## 1. Introduction

Hereditary spastic paraplegias (HSPs) is a class of heterogeneous neurodegenerative diseases affecting 1.8 in 100,000 people (1). They present with bilateral lower limb spasticity, hyperreflexia, and the extensor plantar reflex (2). Pathologically, HSPs are characterized by length-dependent retrograde axonal degeneration of the corticospinal and posterior tracts (2). They are classified as either pure (with symptoms confined to lower limb spasticity and weakness) or complex (additional neurological symptoms such as seizures, cognitive impairment, ataxia, deafness, extrapyramidal disturbances, and peripheral neuropathies) (3). Advances in next-generation genome and exome sequencing have led to the identification of more than 80 HSP-related genes (4–6), with multiple modes of inheritance, including autosomal dominant, autosomal recessive, X-linked, and mitochondrial.

HSPs can present at any age, from infancy onwards, with the average age of onset being between 10 and 30 years (6, 7), depending on the causative gene (8). However, there has been little research specifically focused on pediatric-onset HSPs (9–12) and we are unaware of any large-scale analysis. HSP symptoms in children are similar to those of spastic cerebral palsy (13); therefore, the specificity of symptoms is low.

We investigated the genetic diagnostic yield, genetic background, and clinical features to determine the unique characteristics of pediatric-onset HSPs.

## 2. Materials and methods

### 2.1. Study design and participants

This single-center retrospective study analyzed data from children (aged ≤18 years) diagnosed with HSP in Kanagawa Children’s Medical Center, a tertiary pediatric hospital in Japan between January 1, 2012, and December 31, 2021. Patients with bilateral lower-extremity spasticity and an HSP diagnosis were included using a search for “hereditary spastic paraplegia” or “spastic paraplegia” against the institution’s medical records. Those with only lower-extremity spasticity were classified as pure-type, and those with lower-extremity spasticity and other neurological symptoms, such as seizures, cognitive impairment, ataxia, extrapyramidal symptoms, and hearing loss, were classified as complex-type. We excluded patients with systemic diseases, such as metabolic disorders and acquired central nervous system disorders. Patient medical data, including genetic analysis results, family medical history, clinical information (time of onset, modified Rankin Scale for Neurologic Disability [mRS] scores, neurological symptoms, and treatment and management), brain and spinal cord MRI findings, and electrophysiologic study results, were obtained from the institution’s medical records.

### 2.2. Ethics statement

This study was conducted as per the tenets of the 2013 revision of the Declaration of Helsinki. It was approved by the Ethics Committee of Kanagawa Children’s Medical Center (approval number: 2101-5). The parents or guardians of the patients provided written informed consent to the participation of their children and the publication of this study.

### 2.3. Genetic analysis

Results of genetic analysis were obtained from the patient records. We used exome analysis, direct sequencing, or whole-exome sequencing for peripheral blood. For the pure-type with a family history, *SPAST* sequencing was performed first, and exome panel analysis or whole exome analysis was performed for negative cases. For the pure-type without a family history and all complex-type, exome panel analysis or whole exome analysis was performed. Trio analysis including both parents was performed in all cases. No patient underwent mitochondrial sequencing. Exome sequencing was performed using a TruSight One Sequencing Panel and the MiSeq platform (Illumina Inc., San Diego, CA, United States). Exome data alignment, variant calling, and variant annotations were assessed as previously described (14). Whole exome analysis was performed as previously described (15). Sequencing data were analyzed with the Burrows-Wheeler Alignment tool for mapping to a reference sequence, SAMTools for converting a SAM file to a BAM file, Picard for eliminating duplicate sequenced regions, the Genome Analysis Toolkit for variant calling, and ANNOVAR4 or SnpEff5 for variant annotation. Furthermore, to exclude common variants, we used data from the NHLBI GO Exome Sequencing Project,^1^ Genome Aggregation Database,^2^ 1,000 Genomes Project,^3^ Exome Aggregation Consortium, Human Genetic Variation Database,^4^ and jMorp.^5^ Out of all the called variants within exons or ± 10 bp from exon–intron, those registered in the above-mentioned database of common variants were eliminated with criteria of MAF 0.01 or higher. Variants were confirmed as true positives by Sanger sequencing. We used the Integrative Genomics Viewer for data visualization. Copy number variation analysis was performed using the log2-ratio of read depth on each exon as described previously and the eXome-Hidden Markov Model method.

The pathogenicity of the identified variants was evaluated according to the guidelines of the American College of Medical Genetics and Genomics (16). Variants were annotated with reference to the following transcripts: *SPAST* (NM_014946.4), *KIF1A* (NM_001244008.2), *ALS2* (NM_020919.4), *SACS* (NM_014363.6), *L1CAM* (NM_001278116.2), and *ATL1* (NM_015915.5). Microarray testing was performed with an Agilent SurePrint G3 Human CGH Microarray Kit 8 60 K (Agilent Technologies, Inc., Santa Clara, CA, United States), per the manufacturer’s instructions, to identify deletions and duplications (17).

### 2.4. Statistical analysis

Statistical analyses were performed using v.24 of SPSS for Windows (IBM Corp., Armonk, NY, United States) software. Comparisons of patients’ ages at the time of investigation and time of onset, and the mRS scores of children with pure- and complex-type HSPs were made with the Mann–Whitney *U* test. Fisher’s exact test was used to compare genetic diagnostic yields, family histories, brain and spinal cord MRI results, peripheral neuropathies, and intellectual disabilities. All the statistical tests were two-sided. *p-*values of <0.05 were considered statistically significant.

## 3. Results

### 3.1. Study population and their clinical backgrounds

**Figure 1:**
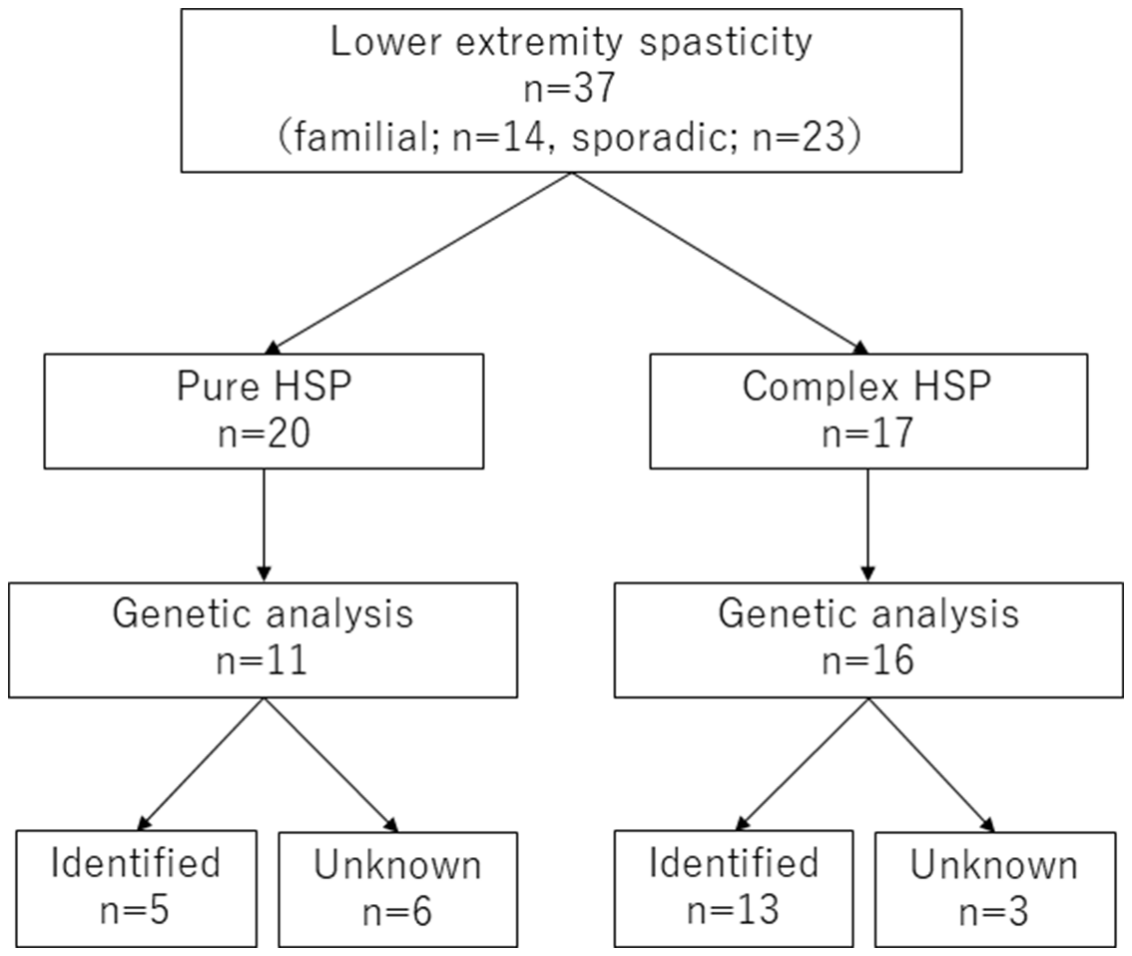
Patient flow chart. HSP, hereditary spastic paraplegia.

Of the 37 patients included in this study, 14 had a family history of HSPs, and 23 were without a clear family history. There were 20 pure-type patients with HSP in the cohort and 17 complex-type. Genetic data were available for 11 of the pure-type and 16 of the complex-type patients. Of these, five (45%) and 13 (81%) patients, respectively, had genetic diagnoses (*p* = 0.11) (Figure 1).

The patients had a median age of 11 years (interquartile [IQR] range, 5–13 years) at the time of investigation, and the median age of onset was 18 months (IQR 13–33). These did not differ significantly between the children with pure-type and complex-type HSPs (Table 1). The incidence of brain MRI abnormalities was significantly higher in children with complex-type than pure-type HSPs. None of the children with pure-type, and only one child with complex-type, HSP had any spinal cord abnormality. Among the 24 patients who underwent electrophysiologic testing, peripheral neuropathy was confirmed in seven (29%) overall, with no significant difference between the pure- and complex-type patients. The mean overall mRS score was 2.8 (±1.2), and the mean in complex-type patients was higher than that of pure-type patients (3.5 [±1.0] vs. 2.1 [±0.9], *p* < 0.001). Of the 17 children with complex-type HSPs, 13 (76%) had an intellectual disability. The most frequent neurological manifestations of complex-type HSPs were dystonia and epilepsy.

**Table 1:** Clinical backgrounds of pediatric patients with hereditary spastic paraplegia in this study.

	Total (*n* = 37)	Pure-type (*n* = 20)	Complex-type (*n* = 17)	*p*-value
Age at investigation (years)	11 (IQR, 5–13)	11 (IQR, 5.75–13.25)	9 (IQR, 5–13)	0.72
Age at onset (months)	18 (IQR, 13–33)	18 (IQR, 15.5–48)	16 (IQR, 12–26)	0.11
Family history	13/37 (35%)	8/20 (40%)	5/17 (29%)	0.73
Brain MRI abnormality	12/35 (34%)	1/19 (5%)	11/16 (69%)	<0.001
Spinal MRI abnormality	1/17 (6%)	0/11 (0%)	1/6 (16.7%)	0.35
Neuropathy	7/24 (29%)	4/14 (29%)	3/10 (30%)	1.00
Modified Rankin Scale for a neurological disability	2.8 ± 1.2	2.1 ± 0.9	3.5 ± 1.0	<0.001
Intellectual disability	13 (35%)	0 (0%)	13 (76%)	<0.001
Other neurological symptoms	–	–	Dystonia 4 (24%)	–
			Epilepsy 3 (18%)	
			Ataxia 2 (12%)	
			Hypotonia 2 (12%)	
			Dysarthria 2 (12%)	
			Eye movement disorder 1 (6%)	
			Nystagmus 1 (6%)	
			Hearing loss 1 (6%)	

The most common treatment was rehabilitation therapy, followed by botulinum toxin type A administration, and surgical treatment (including lower limb tendon lengthening and muscle dissection) (Table 2). Baclofen or levodopa was prescribed to four patients (11%) to treat spasticity and dystonia, respectively.

**Table 2:** Treatments for the pediatric hereditary spastic paraplegia patients in this study.

	Total (*n* = 37)	Pure-type (*n* = 20)	Complex-type (*n* = 17)
Rehabilitation	29 (78%)	13 (65%)	16 (94%)
Botulinum toxin type A	8 (22%)	4 (20%)	4 (24%)
Surgical treatment	3 (8%)	2 (10%)	1 (6%)
Other medical treatment	4 (11%)	1 (5%) baclofen	2 (12%) baclofen
			1 (6%) levodopa

### 3.2. Genetic background

**Figure 2:**
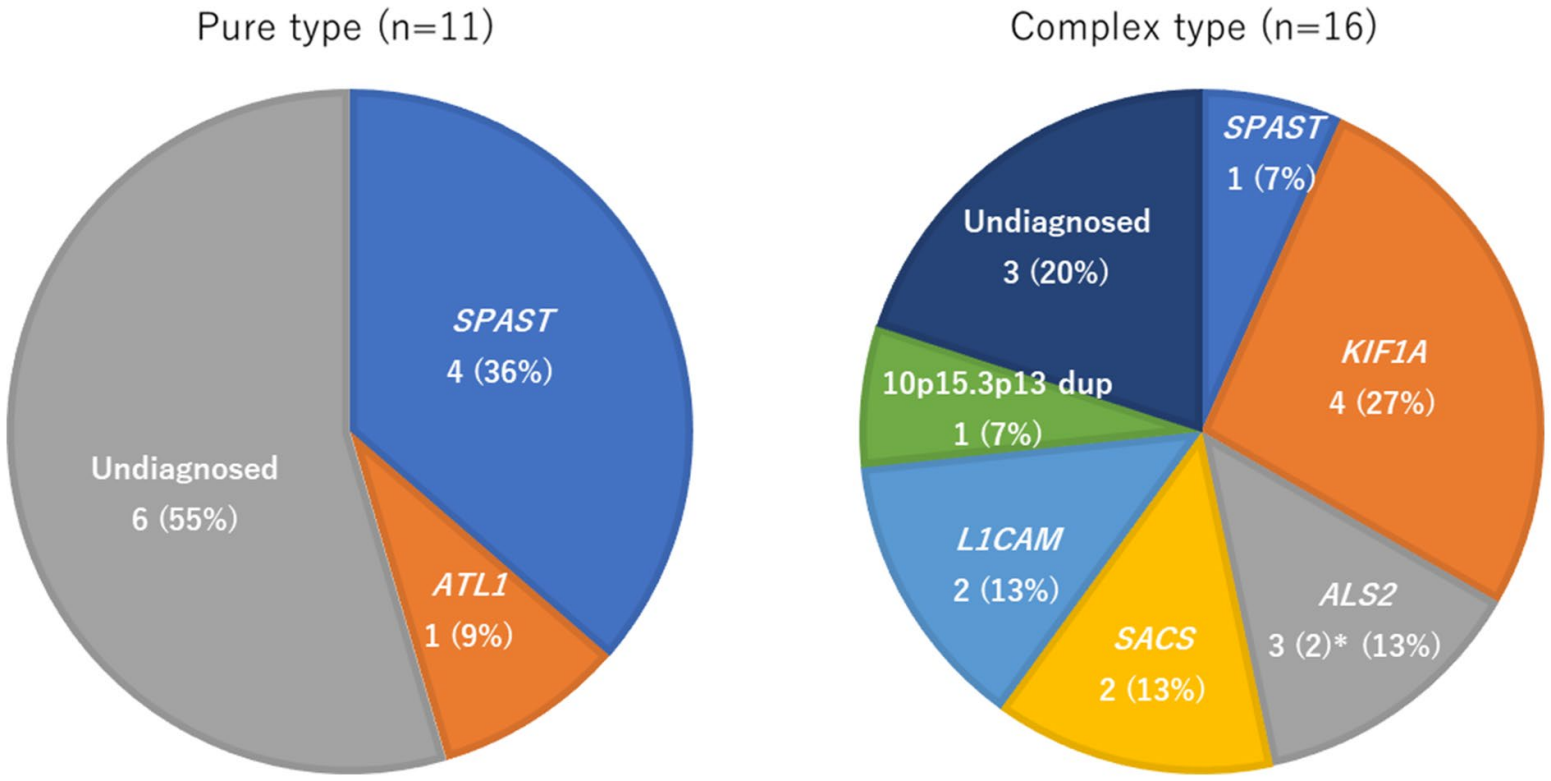
Frequency of genetic factors in pure and complex-type hereditary spastic paraplegia. * Two of the three cases of *ALS2* were monochorionic diamniotic twins, so the frequency of *ALS2* was calculated with *n* = 2.

Figure 2 and Table 3 show the identified causative genes and pathogenic variants. *SPAST* variants were seen in four pure-type and one complex-type HSP. *ALT1* occurred only in pure-type HSPs. *KIF1A*, *ALS2*, *SACS*, and *L1CAM* variants were found only in complex-type HSPs. The 10p15.3p13 duplication was found in one patient with complex-type HSP.

**Table 3:** Genetic and clinical information of each pediatric hereditary spastic paraplegia patient for whom a genetic cause was identified.

	Gene	Variant (coding DNA)	Variant (protein)	ACMG guideline	Inheritance	The type of HSP	Family history	Age at onset (months)	Age at investigation (years)	mRS	Neuropathy	Brain MRI	Intellectual disability	Other neurological symptoms
1	*SPAST*	c.1168A > G	p.(Met390Val)	Likely pathogenic	AD The mother affected	Pure	Yes	17	11	4	No	Normal	None	None
2	*SPAST*	c.1245 + 1G > A	–	Pathogenic	AD The father affected	Pure	Yes	84	13	2	NA	NA	None	None
3	*SPAST*	c.1250G > A	p.(Gly417Glu)	Pathogenic	AD *de novo*	Pure	No	18	5	3	No	Normal	None	None
4	*SPAST*	c.870G > A	p.(Lys290 =)	Uncertain significance	AD The father affected	Pure	Yes	18	9	1	NA	Normal	None	None
5	*SPAST*	c.1684C > T	p.(Arg562*)	Pathogenic	AD The mother affected	Complex	No	10	5	4	NA	White matter volume loss Abnormal signal in white matter	Severe	Hearing loss
6	*KIF1A*	c.308A > G	p.(Lys103Arg)	Likely pathogenic	AD *de novo*	Complex	No	10	12	4	NA	Progressive cerebellar atrophy	Severe	Epilepsy
7	*KIF1A*	c.31C > T	p.(Arg11Trp)	Pathogenic	AD *de novo*	Complex	No	18	5	3	NA	Cerebellar atrophy	Severe	None
8	*KIF1A*	c.761G > A	p.(Arg254Gln)	Pathogenic	AD *de novo*	Complex	No	14	14	4	NA	Periventricular heterotopia Progressive cerebellar atrophy	Mild	None
9	*KIF1A*	c.773C > T	p.(Thr258Met)	Pathogenic	AD *de novo*	Complex	No	18	13	3	NA	NA	Mild	None
10	*ALS2*	c.470G > T c.2325_2326del	p.(Cys157Phe) p.(Phe778Leufs*3)	Likely pathogenic Pathogenic	AR Parent carriers	Complex	yes	12	5	4	No	Normal	None	Dystonia Dysarthria Upper limb spasticity
11	*ALS2*	c.470G > T c.2325_2326del	p.(Cys157Phe) p.(Phe778Leufs*3)	Likely pathogenic Pathogenic	AR Parent carriers	Complex	Yes	12	5	4	No	Normal	None	Dystonia Dysarthria Upper limb spasticity
12	*ALS2*	c.1620delG c.4818_4819insAA	p.(His541Thrfs*13) p.(Tyr1607Asnfs*12)	Pathogenic Pathogenic	AR parent carriers	Complex	No	18	11	5	No	Normal	Mild	Dystonia Dysarthria Eye movement disorder
13	*SACS*	c.12359 T > G c.938_939del	p.(Leu4120*) p.(Val313Alafs*11)	Pathogenic Pathogenic	AR Parent carriers	Complex	Yes	16	16	2	Yes	Cerebellar atrophy T2-hypointensity in pons	None	Ataxia
14	*SACS*	c.12359 T > G c.938_939del	p.(Leu4120*) p.(Val313Alafs*11)	Pathogeni Pathogenic	AR Parent carriers	Complex	Yes	13	6	2	yes	T2-hypointensity in pons	None	Ataxia
15	*L1CAM*	c.3311C > A	p.(Thr1104Asn)	Uncertain significance	XR Mother carrier	Complex	Yes	10	12	4	NA	Hydrocephalus White matter volume loss	Severe	Epilepsy Hypotonia Nystagmus
16	*L1CAM*	c.196C > T	p.(Gln66*)	Pathogenic	XR Mother carrier	Complex	no	11	9	4	NA	Severe hydrocephalus	Severe	Epilepsy
17	*ALT1*	715C > T	p.(Arg239Cys)	Likely pathogenic	AD The mother affected	Pure	Yes	33	5	2	No	Normal	None	None
18	10p15.3p13 duplication	–	arr[GRCh37] 10p15.3p13(193492-12,539,662)x3		–	Complex	No	33	6	2	NA	Thin corpus callosum	Mild	None

ACMG, American College of Medical Genetics and Genomics; AD, autosomal dominant, AR, autosomal recessive; HSP, hereditary spastic paraplegia; mRS, modified Rankin Scale; NA, not available; XR, X-linked recessive.

The inheritance of *SPAST* was autosomal dominant and all variants found have been previously reported. The variants were diverse and included missense, nonsense, and intronic mutations, and one silent mutation. Four patients had a family history of HSPs. The only complex-type patient with a *SPAST* variant also had a *SOX10* variant, which is the causative gene in Waardenburg syndrome. The patient also had unilateral heterochromia iridis, severe deafness, and Hirschsprung disease, all of which are symptoms of Waardenburg syndrome (18). The age of onset and severity of HSPs varied among the patients. In all patients with missense mutations, HSP onset occurred in the first year of life and there was a high degree of spasticity (mRS score 3–4). In a patient with silent mutation, the onset of HSPs was at 18 months, with mild symptoms, and an mRS score of 1.

All *KIFIA* variants showed autosomal dominant inheritance and were *de novo*. None of the four patients with this variant had a family history of HSP. One patient had a previously unreported missense mutation (308A > G) and the other three had previously reported missense mutations. All patients with *KIFIA* variants had complex-type HSPs. Cerebellar atrophy was observed in the three patients with MRI results, with two of these having undergone serial evaluations that had shown their HSPs to be progressive. The age of onset in all patients was ≤1 year and all had severe mRS scores (3, 4), and intellectual disabilities.

The inheritance of *ALS2* variants was autosomal recessive. Two of the three complex-type patients with *ALS2* variants were monochorionic diamniotic twins and shared previously unreported missense (c.470G > T) and frameshift (2325_2326del) mutations associated with their HSPs (Table 3). The same codon mutation has been reported in a patient with infantile-onset ascending spastic paraplegia (19). In addition to the spasticity of the lower extremities, symptoms included spasticity in the upper extremities and dysarthria. There was no intellectual disability. The third patient had a previously unreported frameshift mutation for both alleles, spasticity of the upper extremities, eye movement disorder, and dysarthria. Both phenotypes matched those of infantile-onset ascending HSP caused by an *ALS2* variant (20, 21). All three patients had dystonia.

Inheritance of variants in *SACS* was autosomal recessive. The two patients with these variants were siblings with compound heterozygous unreported nonsense and frameshift mutations (Table 3). Both had pyramidal and cerebellar symptoms, and peripheral neuropathy, which are the three hallmarks of Charlevoix–Saguenay autosomal recessive spastic ataxia caused by *SACS* variants (22). Both exhibited pontine linear low-intensity lesions on T2-weighted MRI. This is characteristic of patients with *SACS* variants (23). Neither patient exhibited any deterioration in their condition and both were able to walk unaided.

Inheritance of *L1CAM* variants was X-linked recessive. This is the causative gene in X-linked hereditary hydrocephalus and X-linked spastic paraplegias (24). The age of onset in the two affected patients was 10–11 months and both had congenital hydrocephalus and severe motor dysfunction. One of the two had undergone surgical periarticular muscle dissection of the hip for severe spasticity. Both patients had severe intellectual disabilities and epilepsy.

The *ATL1* variant occurred in one patient and showed autosomal dominant inheritance. This was diagnosed after the mother was affected. The child had pure-type HSP with mild spasticity and an mRS score of 2.

The 10p15.3p13 duplication we found was a previously unreported 12.3 Mb duplication. The affected patient had a mild intellectual disability. The patient’s brain MRI results showed thinning of the corpus callosum. The age of onset was 33 months, which was late compared to the onset ages of the other patients in this study. The patient’s mRS score was two, and the spasticity was mild.

## 4. Discussion

In this retrospective analysis, we found a high genetic diagnostic yield among patients with pediatric-onset HSP, especially among those with complex-type HSPs. *SPAST* and *KIF1A* were the most common causative genes in pure-type and complex-type HSPs, respectively. The latter has not been previously reported.

In adults with HSPs, the overall genetic diagnostic yield has been estimated as 29–58% for pure-type HSPs (5, 8, 25) and 49% for complex types (26). Our findings reflected this pattern, but our values were substantially higher, with a 67% yield overall, and an 81% yield among children with complex-type HSPs.

Overall, 62% of the patients in our cohort had no clear family history. This is a higher incidence than has been reported in adult-onset HSP patients (13–40%) (5). Pathogenic variants inherited in an autosomal dominant manner tended to be *de novo*, as was the case for all of the *KIF1A* variants identified. Thus, in children with relevant symptoms, pediatric-onset HSPs should be suspected even in the absence of family history.

We found *SPAST* to be the most prominent causative gene in pure-type HSP, having a diagnostic genetic yield of 36%. This roughly corresponds to the previously reported yields in both adult and child-onset HSPs (5, 8). In concordance with the findings of previous research, we found varying ages of onset and HSP severity in patients with *SPAST* variants (8, 27). *ATL1* has previously been reported as common in pediatric-onset HSP (28); however, there was only one child with an *ATL1* variant in this study.

We found *KIF1A* to be the most frequent causative gene among children with complex-type HSPs. This is contrary to previous reports, including those with adult-onset HSP patients, which have found *SPG11* to be the most common causative gene in complex-type HSPs (26). In the present study, there were no patients with *SPG11* variants. The phenotypic spectrum of *KIF1A* includes HSP, ataxia, neuropathy, developmental delay/intellectual disability, optic nerve atrophy, cerebellar atrophy, and hereditary sensory autonomic neuropathy (29). HSP, especially the complex type, is the most frequent phenotype and is characterized by high predispositions for cerebellar atrophy, epilepsy, peripheral neuropathy, and ataxia (30). *KIF1A* variants have previously been found to account for 6–7% of HSPs with identified genetic causes (31). Considering that *KIF1A*-associated HSPs predominantly manifest in childhood (29–31), it could be regarded as one of the major causative genes in pediatric-onset HSPs. All three patients in our study with *KIF1A* variants who underwent brain MRI scans were found to have cerebellar atrophy, and two of these patients had serial scans showing progression. The incidence of cerebellar atrophy in *KIF1A*-related diseases has been reported to range between 35–90% (29, 30), with possible progression over time (32). Such imaging findings could help differentiate *KIF1A*-associated HSPs.

Among the patients with *ALS2, SACS,* and *L1CAM* variants in our cohort, several exhibited clinical symptoms characteristic of spastic paraplegia. All three patients with pathogenic variants in *ALS2* had upper extremity spasticity and dysarthria, which are typical characteristics of infantile-onset ascending HSP. They all also had dystonia. Multiple studies have reported dystonia in infantile-onset ascending HSP and juvenile amyotrophic lateral sclerosis associated with *ALS2* variants (19, 33, 34). Based on the previous reports on *SACS* variants (22, 23), our patients had pyramidal tract symptoms, cerebellar symptoms, peripheral neuropathies, and typical findings of pontine linear hypointense lesions on T2-weighted brain MRI. Therefore, these features could be useful as confirmatory indicators of this genetic cause. In boys with congenital hydrocephalus, severe motor and intellectual disabilities, and epilepsy, *L1CAM* variants should be suspected.

Our identification of the 10p15.3p13 duplication as a causative factor in spastic paraplegia was a unique finding, and there are no known genes associated with spastic paraplegia in the same region. However, the *ZMYND11* gene in the region of the 10p15.3p13 duplication has previously been identified as a causative gene in intellectual disabilities, autism, epilepsy, hypotonia, and dysmorphism (35, 36). Therefore, the potential association between this gene and the spastic paraplegia phenotype requires further investigation.

Table 4 shows a comparison of the causative genes identified in previous reports of pediatric-onset HSPs and this study. *SPAST, ALT1*, and *SPG11* tend to be relatively frequent, but other causative genes are diverse, and most of them are rare pathogenic variants with 1–2 cases each. In our cohort, the frequency of *SPAST* and *KIF1A* was high, but the others were various pathogenic variants in 1–2 cases each. The causative genes of pediatric-onset HSPs are presumed to be diverse, however, reports on the causative genes of pediatric-onset HSPs are limited and more cases are needed to describe trends.

**Table 4:** Causative genes in the previous literature on pediatric-onset hereditary spastic paraplegia.

Author, year	Number of study subjects (*n*)	Genetic diagnostic yield (*n*), *n* (%)	Methods of genetic analysis	Identified as a causative gene (*n*, %)
Present study	37	18/27 (67%)	Direct sequencing (*SPAST*) disease-associated panels whole-exome sequencing microarray testing	*SPAST* (5, 28%), *KIF1A* (4, 22%), *ALS2* (3*, 17%), *SACS* (2, 11%), *L1CAM* (2, 11%), *ATL1* (1, 6%), 10p15.3p13 duplication (1, 6%)
Travaglini et al. (9)	47	29/47 (62%)	Disease-associated panels MLPA screening (*SPAST*) SNP array analysis	*SPG11* (7, 24%), *SPAST* (6, 21%), *ALT1* (2, 7%), *ALS2* (2, 7%), *ERLIN2* (2, 7%), *POLR3A* (1, 3%), *FA2H* (1, 3%)*, DDHD2* (1, 3%)*, ATP2B4* (1, 3%)*, ENTPD1* (1, 3%)*, CAPN1* (1, 3%)*, ADAR1* (1, 3%)*, RNASEH2B* (1, 3%)*, TUBB4A* (1, 3%)*, KIF1A* (1, 3%)
Schiavoni et al. (10)	47	17/47 (36%)	Disease-associated panels whole-exome sequencing	*ATL1* (4, 24%), *SPAST* (3, 18%), *FA2H* (2, 12%), *REEP1* (1, 6%)*, KIF5A* (1, 6%)*, KIF1A* (1, 6%)*, ITPR1* (1, 6%)*, CYP2U1* (1, 6%)*, DDHD2* (1, 6%)*, RNASEH2B* (1, 6%)*, L1CAM* (1, 6%)
Giordani et al. (11)	106 (83 families)	68/106 (64%) (50/83 (60%) families)	Direct sequencing (*SPAST*) disease-associated panels	*SPAST* (11, 22%), *ALT1* (8, 16%), *SPG11* (5, 10%), (Argininemia (3, 6%)), *PLP1* (3, 6%), *GBA2* (3, 6%), *ZFYXE26* (2, 4%), *ENTPD1* (2, 4%), *SPG7* (2, 4%), *SPOAN* (2, 4%), *ALS2* (1, 2%), *DADA2* (1, 2%)*, DRD,* (1, 2%) *KIF5A* (1, 2%)*, KIF1A* (1, 2%)*, REEP1* (1, 2%)*, CYP7B1* (1, 2%)*, REEP2* (1, 2%)*, KIAA0196* (1, 2%) (families)
Panwala et al. (12)	16	14/16 (88%)	Disease-associated panels whole-exome sequencing	*SPAST* (3, 21%), *MARS* (2, 14%), *KIF1A* (2, 14%), *KIF5A* (1, 7%), *SACS* (1, 7%), *SPG7* (1, 7%), *REEP1* (1, 7%), *PNPT1* (1, 7%), *MT-ATP6* (1, 7%), *ATL1* (1, 7%)

^*^Two of the three cases of ALS2 were monochorionic diamniotic twins.

This study was limited by its retrospective, single-center design, and its small sample size. Patient selection is based on medical record searches and may not include all patients. Owing to the small sample size, it may be difficult to generalize the frequencies of causative genes. Moreover, the patients in our sample were all Japanese, limiting the generalizability of the genetic background results, which may include racially specific traits.

## 5. Conclusion

Our findings suggest that pediatric-onset HSPs are more likely to manifest in a sporadic form. We found it to have a high genetic diagnostic yield. *SPAST* and *KIF1A* were the most common causative genes among children with pure-type and complex-type HSPs, respectively. In complex-type HSPs, the clinical symptoms may help differentiate between causative pathogenic variants such as *ALS2, SACS*, and *L1CAM*. Further research with larger cohorts is needed to investigate the clinical manifestations, genetic workup, and genetic differences between pediatric and adult-onset cases.

## Data availability statement

The datasets presented in this study can be found in online repositories. The names of the repository/repositories and accession number(s) can be found in the article/Supplementary material.

## Ethics statement

The studies involving human participants were reviewed and approved by ethics committee of Kanagawa Children’s Medical Center (approval number: 2101–5). Written informed consent to participate in this study was provided by the participants’ legal guardian/next of kin. Written informed consent was obtained from the individual(s), and minor(s)' legal guardian/next of kin, for the publication of any potentially identifiable images or data included in this article.

## Author contributions

The study conception and design were by AI. Genetic analysis was performed by TK, YE, AF, HS, NM, and KK. Patient diagnoses and follow-ups were performed by AI, YT, MT, and TG. Data collection was by AI. The first draft of the manuscript was written by AI. The manuscript was reviewed and revised by MT and TG. All authors contributed to the article and approved the submitted version.

## Funding

This work was supported by the Japan Agency for Medical Research and Development (grant numbers: JP22ek0109486, JP22ek0109549, and JP22ek0109493 (NM), and JP20ek0109301 (KK)); JSPS KAKENHI (grant numbers: JP20K17936 and JP22K15901 (AF), JP20H03641 (HS), JP18K07864 (YE), and JP20K08270 (KK)); and the Takeda Science Foundation (NM).

## Conflict of interest

The authors declare that the research was conducted in the absence of any commercial or financial relationships that could be construed as a potential conflict of interest.

## Publisher’s note

All claims expressed in this article are solely those of the authors and do not necessarily represent those of their affiliated organizations, or those of the publisher, the editors and the reviewers. Any product that may be evaluated in this article, or claim that may be made by its manufacturer, is not guaranteed or endorsed by the publisher.

## Footnotes

^1^https://evs.gs.washington.edu/EVS/

^2^https://gnomad.broadinstitute.org/

^3^https://www.internationalgenome.org/

^4^http://www.hgvd.genome.med.kyoto-u.ac.jp/

^5^https://jmorp.megabank.tohoku.ac.jp/
